# Burden of Cancer Due to Cigarette Smoking and Alcohol Consumption in Korea

**DOI:** 10.3390/ijerph19063493

**Published:** 2022-03-15

**Authors:** Yoon-Sun Jung, Seok-Jun Yoon

**Affiliations:** 1 Institute for Future Public Health, Graduate School of Public Health, Korea University, Seoul 02841, Korea; sunnyaurora@nate.com; 2 Department of Preventive Medicine, Korea University College of Medicine, Seoul 02841, Korea

**Keywords:** burden of disease, disability-adjusted life years, population attributable fraction, Korea, risk factors

## Abstract

This study aimed to estimate the burden of cancer in Koreans attributable to smoking and alcohol consumption using disability-adjusted life years and population attributable fractions. We estimated the burden of 12 cancers due to simultaneous and independent smoking and alcohol exposure in Koreans aged ≥40 years. In men, the cancer burden attributable to the combined risk factors, smoking alone, and alcohol consumption alone were 9.5, 14.8, and 6.1%, respectively; the corresponding values for women were 1.1, 2.5, and 2.7%, respectively. In men, tracheal, bronchial, and lung cancers were the most common cancer types. The disease burden may have been reduced by 16.8, 32.3, and 4.1% in the absence of the combined risk factors, smoking alone, and alcohol consumption alone, respectively. Our findings suggest that risk factor-based intervention may have the greatest preventative effect for lung cancer among all cancers in men. Our real-world data methodology could provide further evidence-based methods to explore and facilitate effective health promotion interventions for specific target groups and may lay the foundation for the establishment of healthcare services according to population subgroups or regional characteristics.

## 1. Introduction

Some diseases are associated with past or present exposure to certain health risk factors; thus, prevention and management of known risk factors are important from a public health perspective. Preventing disease and injury through policies and primary care interventions aiming to improve the control of modifiable risk factors, such as smoking, is a key challenge in public health. Therefore, quantifying the exposure levels of leading risk factors and determining their effects on human health are important research areas to identify the sectors of public health where current efforts are inadequate [1,2]. To this effect, studies quantifying the risk factors associated with each disease at a population level and determining their effects on society are warranted in order to facilitate the development of strategies for health risk factor interventions that can improve health at the population level.

In everyday life, it is more common for people to engage in a combination of two or more existing risk factors, rather than only one health risk behavior, for example, active cigarette smoking and high-risk alcohol consumption or a combination of active smoking and obesity. In Korea, 66.0% of men and 43.9% of women aged ≥30 years have at least two combined health risk factors [3], which synergistically increase disease risk beyond the simple sum of the negative effects of each risk factor alone [4,5]. Moreover, individuals with combined risk factors are more likely to have poor subjective health and to report feeling stressed or depressed [6]. These findings suggest that to develop a disease prevention and management strategy through healthy living practices, an integrated form of risk factor management may be required, rather than one focused on managing individual health risk factors [3].

Drinking is a social behavior influenced by various environmental factors such as availability, public policies, and acceptance of alcohol use. In Korea, drinking is characterized by a pattern of going back and forth between social drinking and problem drinking [7]. Owing to Korea’s remarkable economic growth that brought about widespread social changes, gender became an important variable affecting smoking patterns. Korea’s rapid industrialization and urbanization resulted in changes to women’s socioeconomic status, including making women more likely to smoke than before [8]. Meanwhile, in Korea, men remain subject to mandatory military service, which has been associated with initiating and maintaining smoking behavior [8].

Several national and international studies have demonstrated that smoking and alcohol consumption are major risk factors for cancer development [9,10,11,12,13,14,15,16,17,18,19,20,21]. Although the combined effect of smoking and alcohol consumption on cancer incidence and mortality may be significant, few studies have investigated these effects in the Korean population. Cigarette smoking and alcohol consumption are physiologically, psychologically, and socially related [22,23,24]; thus, an individual can engage in both behaviors. Alcohol consumers have higher smoking rates than non-consumers, and smokers consume more alcohol than non-smokers [22]. Therefore, the magnitude of the combined effects of smoking and alcohol consumption on health needs to be determined. In addition, a comprehensive and multidimensional intervention should be developed to prevent smoking and alcohol consumption, which can be modified through behavioral changes.

The Global Burden of Disease (GBD) study used a standardized and comprehensive methodology to estimate country-specific burden of disease, including mortality rates, attributable to a single health risk factor and a combination of risk factors [1]. Several studies in Korea have estimated the national burden of disease since the late 1990s [25]; these estimates continue to be updated, with refinements to measurement methodologies for the disease burden of all diseases, including cancer [26,27,28]. The Korean National Burden of Diseases (KNBD) study used national health insurance claims data to expand the measurement scope by utilizing domestic data sources and advanced methodologies [26]. With the availability of sufficient data and a validated KNBD measurement methodology, several studies have examined the disease burden attributable to health risk factors, focusing on a single health risk factor or a single disease [29,30,31]. However, these previous studies have only considered other health risk factors as covariates, and no previous study has examined the combined effects of risk factors on disease burden.

Quantifying the burden of disease attributable to specific risk factors in a comprehensive and comparable way may provide a foundation for public health policy design. To this end, the contribution of risk factors to disease burden can be measured by population attributable fractions (PAFs), which integrate information of “dangerousness” and “prevalence” of risk factors, supporting decision-making on population health protection and disease prevention.

Therefore, this study aimed to quantitatively measure the cancer burden attributable to smoking and alcohol consumption in Koreans by analyzing domestic data sources. In addition, to provide further information for formulating broader and more meaningful prevention strategies, the combined effect of the two health risk behaviors was evaluated.

## 2. Materials and Methods

### 2.1. Disease Selection and Calculation of Disability-Adjusted Life Years (DALYs)

Smoking and alcohol consumption were analyzed separately and as combined risk factors. In addition, based on previous meta-analyses [9,10,11,12,13,14,15] and epidemiological studies [16,17,18,19,20,21], cancer types according to specific sites associated with both risk factors were selected as follows: esophageal cancer (C15; D001); stomach cancer (C16; D002; D371); liver cancer (C22; D015; D376); laryngeal cancer (C32; D020; D380); tracheal, bronchial, and lung cancers (C33; C34; D021; D022; D381); colon and rectal cancers (C18; D010; D374; C20; D012; D375; C19; C21; D013; D373; D011); mouth cancer (C00–C08); nasopharyngeal cancer (C11); cancer of other parts of the pharynx and oropharynx (C09; C10; C12; C13); gallbladder and biliary tract cancer (C23; C24); pancreatic cancer (C25); and bladder cancer (C67; D090; D414). A total of 12 cancer types were included in the analysis, which are some of the cancer diseases defined from the disease classification system used in the KNBD study [26,27]. As the Korean national cancer screening program targets adults aged ≥40 years [32], our study population was limited to this age group.

To investigate cancer burden in Korea, the number of DALYs for cancers in Koreans aged ≥40 years as of 2018 was calculated. This included calculating the following components using the most recent KNBD measurement methodology: years of life lost (YLLs) and years lived with disability (YLDs) [26]. For YLL calculations, cause-of-death data, complete life tables, and mid-year population were used based on the resident registration published by Statistics Korea. For YLD calculations, National Health Insurance claims data provided by the National Health Insurance Service (NHIS) and Korean National Hospital Discharge In-Depth Injury Survey data published by the Korea Disease Control and Prevention Agency were used. Additional details on the specific methodology used to estimate DALYs in Koreans are described in KNBD publications [26,27].

### 2.2. Estimation of PAFs of Cancers due to Smoking and Alcohol Consumption

To estimate the DALYs of cancers attributable to the two selected risk factors, PAFs were estimated by combining the relative risk (RR) and rate of exposure to risk factors (Pe) using Levin’s formula for PAF [33].

(1) $$\mathrm{PAF}=\frac{\mathrm{Pe}\left(\mathrm{RR}-1\right)}{1+\mathrm{Pe}\left(\mathrm{RR}-1\right)}$$

RR indicated the relationship between each risk factor and health outcomes, cancer incidence, or mortality rates. PAFs represented the fraction of disease and injury in a population due to exposure to a risk factor (i.e., the contribution of a risk factor to the proportion of disease and injury) [33,34]. The NHIS-National Sample Cohort (NHIS-NSC) database was used to calculate the exposure rate and the RR of the risk factors constituting the PAFs. The NHIS-NSC is a population-based cohort established by the NHIS in South Korea, which comprises 2% (approximately 1 million people) of the total eligible Korean population. The NHIS-NSC database holds representative population-based cohort data and consists of four databases on insurance eligibility, medical treatments, medical care institutions, and general health examinations [35]. In NHIS-NSC, diagnosis information according to the Korean Standard Classification of Diseases (KCD) code was recorded from 2002 to 2015.

To accurately estimate the incidence of cancer using the cohort data, a washout period of 2 years (2002–2003) was used to exclude all cancer patients diagnosed with the cancers of interest within this period. Subsequently, the enrollment date was set as an individual’s 2004 or 2005 health check-up date. Since the entire population of Korean adults aged ≥40 years can apply for the general health screening program at least once every 2 years for the entire population of Korean adults aged ≥40 years [36], the range of enrollment dates was set to two years. For those who underwent medical checkups in both 2004 and 2005, the results from 2005 were used.

To calculate the exposure rates for smoking, alcohol consumption, and combined smoking and alcohol consumption in men and women aged ≥40 years, frequency analyses were performed using the 2004 and 2005 NHIS-NSC databases. Individuals who responded, “I currently smoke” were categorized into the smoking exposure group, and individuals who indicated an alcohol consumption frequency of “>3–4 times a week” and alcohol volume consumption per event of more than “a bottle of soju” were categorized into the alcohol exposure group. Soju is the most popular Korean liquor drink that generally contains 17.5% alcohol per bottle (360 mL). The study aimed to match the operational definition of high-risk alcohol consumption used by Korea Health Statistics as closely as possible. In addition, individuals who met both the smoking and alcohol exposure definitions were categorized into a smoking and alcohol consumption (SA) group, indicating exposure to both risk factors. Finally, individuals who did not actively smoke cigarettes and did not consume alcohol were categorized into a nonsmoking and non-alcohol consumption (NSNA) group.

To determine the relationship between exposure to risk factors and cancer incidence or death, RRs were calculated. Individuals who participated in the national health screening program in 2004 or 2005 and whose data were used for RR calculation were divided into risk factors-exposed and non-exposed groups. Individuals with a medical record of target cancer prior to their 2004 or 2005 medical examination were excluded from the analysis.

To calculate the RR between risk factors and the incidence or mortality related to each cancer, the Cox proportional hazards model was used, and hazard ratios (HRs) and 95% confidence intervals (CIs) were estimated. The operational definition of smoking in the calculation of the HRs was applied differently from that used in estimating the exposure rate of smoking, as there is a lag-time of ≥20 years between exposure to smoking and disease incidence [37]. Current smokers with a smoking duration of 20–29 years or >30 years were included in the smoking exposure group for HR calculations. International Classification of Diseases 10th edition codes were used to define the occurrence of an event.

The start date of the follow-up period was based on each individual’s medical examination date in 2004 or 2005. Survival time for each participant in our study was defined as the length of time from the date of the medical examination to the date of the earliest occurrence of one the following: cancer diagnosis, cancer death, loss to follow-up, death, or 31 December 2015, which was the maximum traceable time in the NHIS-NSC database. If HR calculations were not possible owing to an insufficient number of individuals in one sex group, sex-adjusted values for the same disease were substituted.

When the estimated HR value was <1 for risk factors, an HR of 1 was used instead for PAF calculation. Previous studies [9,10,11,12,13,14,15,16,17,18,19,20,21] have shown that smoking and alcohol consumption are risk factors that increase the incidence of cancer and cardiovascular diseases. All analyses for HR estimation for specific cancers were performed separately according to sex and risk factors. In addition, we tested the statistical significance of the interaction effect by adding the interaction term of smoking and alcohol consumption to the Cox proportional hazard model and confirming a *p*-value of <0.05.

Factors confirmed to be associated with the incidence or mortality of each cancer type were considered as covariates in the analysis model, which included age, residential area, income level, frequency of physical activity, total cholesterol level, fasting blood sugar level, body mass index, blood pressure, family history of cancer, personal history of hypertension, and personal history of diabetes mellitus. In the calculation of HRs, the fit of the Cox proportional hazard model was determined by analyzing Schoenfeld residuals. Meanwhile, covariates that did not satisfy the proportional hazards assumption were excluded from the model. Final adjusted HRs are reported. All analyses were performed using SAS 9.4 (SAS Institute Inc., Cary, NC, USA), and statistical significance was set at 5%.

### 2.3. Burden of Cancer Attributable to Cigarette Smoking and Consuming Alcohol

To estimate the DALYs for a specific cancer type attributable to exposure to each risk factor, we multiplied PAFs for incidence or mortality by YLLs or YLDs and summed attributable YLLs and YLDs according to sex, cause (specific cancer), and risk factor. The equation used to calculate the DALYs for cancers in Koreans attributable to smoking and alcohol consumption was as follows:

(2) $$ADALY_{x}=\left(\underset{d}{\sum }YLL_{d}*PAF_{xdd}\right)+\left(\underset{d}{\sum }YLD_{d}*PAF_{xdi}\right)$$

where *ADALY_x_* was the DALYs attributable to risk factor *x*, *YLL_d_* was YLLs of cancer *d*, *PAF_xdd_* was the fraction of YLL for cancer *d* due to risk factor *x*, *YLD_d_* was YLDs of cancer *d*, and *PAF_xdi_* was the fraction of YLD for cancer *d* due to risk factor *x*. The burden of cancer attributable to each risk factor was equal to the sum of the attributable burdens for each cancer type related to that risk factor.

## 3. Results

### 3.1. National Burden of Cancer in Korea, 2018

Table 1 shows the number of YLLs, YLDs, and DALYs for smoking- and alcohol consumption-related cancers according to sex in Koreans aged ≥40 years in 2018. In men, the number of YLDs for major cancers related to smoking and alcohol consumption was 197,090 person-years, and that of YLLs was 309,363 person-years, constituting the number of DALYs of 506,453 person-years. Based on the number of DALYs, cancer type-specific burden in men ranged from 119,550 (tracheal, bronchial, and lung cancers) to 2560 (nasopharyngeal cancer). In women, the number of YLDs was 93,669 person-years and that of YLLs was 150,461 person-years, constituting the number of DALYs of 244,130 person-years; the corresponding cancer type-specific disease burden ranged from 53,963 (colon and rectal cancers) to 561 (laryngeal cancer) based on the number of DALYs (Table 1).

### 3.2. PAFs of the Incidence and Mortality of Cancers

Table 2 shows the incidence and mortality estimates for specific cancers according to smoking and alcohol consumption among Korean men aged ≥40 years. Smoking and alcohol consumption showed no significant interaction for the incidence or mortality rates in any cancer type. Compared with the nonsmoking and non-alcohol consumption (NSNA) group, the smoking and alcohol consumption (SA) group had an increased risk of esophageal cancer (HR 4.4, 95% CI 2.7–7.3); stomach cancer (HR 1.3, 95% CI 1.1–1.5); liver cancer (HR 1.6, 95% CI 1.3–2.0); laryngeal cancer (HR 3.4, 95% CI 1.9–6.3); tracheal, bronchial, and lung cancers (HR 2.3, 95% CI 1.9–2.7); colon and rectal cancers (HR 1.3, 95% CI 1.1–1.6); and pancreatic cancer (HR 1.6, 95% CI 1.1–2.5). A combination of smoking and alcohol consumption increased the incidence rates of esophageal cancer, liver cancer, and tracheal, bronchial, and lung cancers compared with smoking and alcohol consumption separately.

Compared with the NSNA group, the SA group had an increased risk of mortality due to esophageal cancer (HR 6.1, 95% CI 2.8–13.6); liver cancer (HR 2.2, 95% CI 1.5–3.2); laryngeal cancer (HR 23.4, 95% CI 1.7–313.4); tracheal, bronchial, and lung cancers (HR 3.3, 95% CI 2.5–4.4); colon and rectal cancers (HR 1.7, 95% CI 1.0–2.8); and pancreatic cancer (HR 1.9, 95% CI 1.1–3.3). Of these, the risk of mortality from esophageal cancer and liver cancer due to exposure to combined smoking and alcohol consumption was higher than that due to smoking and alcohol consumption when considered independently (Table 2). Detailed information on the number of cases and follow-up is presented in Supplementary Table S1.

Table 3 shows the HRs of incidence and mortality rates for specific cancer types according to smoking and alcohol consumption in Korean women aged ≥40 years. There was no significant interaction between smoking and alcohol consumption for the incidence or mortality rates associated with any cancer type. Compared with the nonsmoking and non-alcohol consumption (NSNA) group, the smoking and alcohol consumption (SA) group had a significantly increased risk of incidence of liver cancer (HR 3.3, 95% CI 1.0–10.7); tracheal, bronchial, and lung cancers (HR 5.1, 95% CI 2.0–13.0); and pancreatic cancer (HR 6.4, 95% CI 1.3–31.4). The mortality rate of tracheal, bronchial, and lung cancers showed a 13.5-fold increase due to combined smoking and alcohol consumption. In women, as there were many cases where the cancer incidence or death event count was 0, the sex-adjusted value for the same disease was used as a substitute. Therefore, such values are not shown in bold. Detailed information on the number of cases and follow-up is presented in Supplementary Table S2.

Table 4 shows the PAFs attributable to combined smoking and alcohol consumption and those attributable to smoking and alcohol consumption considered independently. In men, the PAF for incidence associated with combined smoking and alcohol consumption was the highest for esophageal cancer (25.5%), followed by laryngeal and lung cancers (19.6 and 11.3%, respectively). In women, the highest incidence attributable to combined smoking and alcohol consumption was 2.0% for pancreatic cancer.

Overall, the PAFs associated with risk factors were higher for cancer mortality than for cancer incidence. Among Korean men aged ≥40 years, combined smoking and alcohol consumption contributed most to cancer mortality for laryngeal cancer (69.2%), followed by esophageal cancer (34.0%) and cancer of other parts of the pharynx and oropharynx (27.7%). In women, combined smoking and alcohol consumption-attributed mortality rate was the highest for laryngeal cancer (4.6%), followed by for tracheal, bronchial, and lung cancers (4.4%) and esophageal cancer (1.9%; Table 4).

### 3.3. Burden of Cancers Attributable to Smoking and Alcohol Consumption according to Sex in Koreans Aged ≥40 Years

Table 5 shows the burden of cancers attributable to smoking and alcohol consumption according to sex and cancer type, measured using DALYs. In 2018, the number of DALYs due to combined smoking and alcohol consumption in Korean men was 48,306 person-years, accounting for 9.5% of the national all-cause DALYs for related cancers. Based on the proportion of the burden of disease due to risk factors, the total DALYs attributable to the combination of smoking and alcohol consumption were highest for laryngeal cancer (2042, 41.3%), followed by for esophageal cancer (5273, 31.2%) and tracheal, bronchial, and lung cancers (20,081, 16.8%).

In contrast, the burden of cancers attributable to smoking and alcohol consumption was relatively smaller in women than in men. In Korean women aged ≥40 years, the total DALYs for cancers related to combined smoking and alcohol consumption and those related to smoking and alcohol consumption considered separately were 2666 (1.1%), 6080 (2.5%), and 6599 (2.7%), respectively. Among the 12 evaluated cancer types, even those with the highest contribution to the burden of cancer did not reach 10.0%.

## 4. Discussion

To our knowledge, this is the first study to quantitatively estimate the burden of cancer attributable to smoking and alcohol consumption in Korean adults aged ≥40 years using DALYs, a summary measure of population health. This study considered the combination of smoking and alcohol consumption to estimate the combined effects of multiple risk factors. Combined smoking and alcohol were found to account for 9.5% (48,306 person-years) of cancer burden in men and 1.1% (2666 person-years) in women. In men, the burden of cancer attributable to smoking alone was 14.8% (75,117 person-years) and that due to alcohol consumption alone was 6.1% (30,934 person-years); corresponding values in women were 2.5% (6080 person-years) and 2.7% (6599 person-years), respectively.

We found that the burden of cancer attributable to combined smoking and alcohol consumption from the highest to the lowest number of DALYs for men was tracheal, bronchial, and lung cancers; liver cancer; stomach cancer; colorectal cancers; and pancreatic cancer. In women, the ranking differed slightly as follows: colorectal cancer; tracheal, bronchial, and lung cancers; stomach cancer; liver cancer; and pancreatic cancer. We compared these findings with the most recently published GBD results for 2019 [2]. As we measured the disease burden in Koreans based on the most recent year, we could calculate the relevant parameters using the available data sources. Although direct comparison with a study by Vos et al. [2] was challenging as their study covered all age groups, the top five cancer types for men were found to be the same in both studies. However, Vos et al. reported that the highest disease burden in women was breast cancer, followed by lung cancer, colorectal cancer, stomach cancer, and liver cancer [2]; these findings were inconsistent with our findings. This discrepancy may reflect the differences in data sources and methodologies used by Vos et al. [2] and the KNBD study. However, although breast cancer ranked high among female cancers [2], this study did not include breast cancer as a target disease. Although we attempted to obtain the RR for breast cancer, a component of the PAF, the NHIS-NSC database defines some diseases, including breast cancer, as sensitive diseases that are subject to data masking.

The risks of cancer incidence and death in individuals who were both active smokers and consumers of alcohol were investigated and compared separately with those of active smokers or alcohol consumers according to sex and specific cancer site. In men who both actively smoked and consumed alcohol, the risks of esophageal cancer; liver cancer; and tracheal, bronchial, and lung cancers were 4.4-, 1.6-, and 2.3-times higher, respectively, than those in non-smokers and non-consumers of alcohol. Furthermore, in men, the HRs of the combined risk factors for death from esophageal cancer and liver cancer were 6.1 and 2.2, respectively. These comparisons were not possible in women because no cancer with significant HR was identified in those who both smoked and consumed alcohol or in those who either smoked or consumed alcohol.

Several studies have compared independent and combined effects of risk factors, focusing on modifiable lifestyle factors. In particular, several studies have estimated the effects of combined smoking and alcohol consumption, focusing on pharyngeal and esophageal cancers. Castellsagué et al. [38] compared the combined and independent effects of smoking and alcohol consumption for esophageal cancer and reported odds ratios (ORs) of 1.95 and 1.75 for only smoking and only consuming alcohol, respectively. However, the OR for combined smoking and consuming alcohol was 8.0, indicating a synergistic interaction between these two risk factors. The risk of developing esophageal cancer has been reported as 23.1- [39], 8.3- [40], or 20.4-times [41] higher in the exposed group who smoked and consumed alcohol than in the non-exposed group. Another study reported that the HR of cancer-related deaths in those who consumed excessive volumes of alcohol and who smoked heavily was 2.9 compared to that in those who did not smoke or consume alcohol [42]. Furthermore, one study found that exposure to either smoking or alcohol consumption was not associated with a significant increase in cancer risk; however, simultaneous exposure to both factors increased the risk of oral and pharyngeal cancers [43]. Hongli et al. [44] compared those who did not smoke cigarettes and consume alcohol and reported that the OR of cigarette smokers and consumers of alcohol for premature death was 3.14. Hart et al. [45] conducted a study involving adults aged 35–64 years and showed that mortality rates due to coronary artery disease, stroke, and respiratory disease were 1.82-, 3.10-, and 8.28-times higher, respectively, in those who actively smoked and consumed alcohol than in those who did not. Overall, behaviors that combine smoking and consuming alcohol can be interpreted as increasing the risk of disease incidence and death beyond the individual impact of each risk factor. The discrepancies in risk estimates among individual studies may be due to differences in measurement methods, such as the definition of the exposure group, follow-up period, and covariates choices.

Several international studies have investigated health effects associated with combinations of various risk factors; however, such studies have not yet been undertaken in Korea, making comparisons with present findings challenging. In Korea, only the association between individual health risk factors and cancer risk has been investigated, wherein studies have evaluated specific age groups, diseases, and regions [46,47,48]. In these studies, to evaluate the health effect of one risk factor, other risk factors were used as control variables. To our knowledge, no previous study has evaluated the effects of risk factors on cancer in the overall population, while considering simultaneous exposure to multiple risk factors.

However, unlike that in men, the impact of risk factors on cancer incidence or mortality rates in women could not be estimated using the research model in our study. Therefore, the PAFs were calculated by substituting the estimated HRs by adjusting for sex. Sex differences in the effects of risk factors on disease onset have been reported [49,50,51]. Biological differences between the sexes may account for some of these differences. However, considering that a population-based dataset was used as the main data source in our study, the lack of sufficient sample size, sex-based differences in risk factor exposure rates, and limited accuracy of survey responses may also explain these differences.

In this study, the burden of lung cancer in men was the highest among the cancers studied, and the proportion of diagnoses due to combined smoking and alcohol consumption was also high (16.8%). These findings indicate that 16.8% of the burden of lung cancer, a major disease that afflicts Korean men, could have been prevented if men aged ≥40 years had refrained from smoking cigarettes and consuming alcohol.

PAFs indicate the proportion of disease risk that could have been prevented if exposure to a risk factor had been eliminated. PAFs are influenced by the prevalence and contribution of certain risk factors to disease development. Therefore, the use of PAFs in this study supports the translation of the present findings to policy decisions. This study examined cancer burden by sex. However, within the entire population, there are also differences in lifestyle in terms of age group, occupation, and residential area. In addition to the level of exposure to risk factors, the health effects of risk factors are likely to differ for each subgroup. In Korea, through the Integrated Health Promotion Program, local governments run projects that help promote healthy living and prevent chronic diseases; these programs are tailored to local resident characteristics and needs. Since 2013, this program has supported local government autonomy, reducing the impact of the state-led top-down approach. Accordingly, it is necessary to proactively develop and promote various projects to improve the health of residents according to local needs. Ultimately, an effective process for targeting population subgroups and health risk factors is needed to maximize the effectiveness of health care services and reduce health inequality. However, studies evaluating interventions in each target group, considering regional characteristics, have yet to be conducted. Although this study focused only on smoking and alcohol consumption and related cancers, the methodology applied in this study may be expanded upon and utilized further in other contexts. Further studies are required to estimate disease burden attributable to regional risk factor distributions, disease risks, and population structures. In addition, efforts to improve the limitations of previous health policies, which focused only on the intervention of a single risk factor, need to be further supported by empirical evidence. This study suggests that the combination of various health risk factors need to be accounted for in any policy development. In-depth studies should be conducted on more diverse diseases to develop specific and diverse intervention strategies for complex health risk factors. Consequently, more effective health care services should be established according to population characteristics.

This study had some limitations. First, owing to limitations in the data sources, the operational definition of high-risk alcohol consumption suggested by Korea Health Statistics could not be accurately matched. Second, the NHIS-NSC data source for PAF estimation designates some categories, including breast cancer, as sensitive diseases that are subject to data masking. Future research may obtain higher quality evidence by calculating the HRs for all diseases related to the risk factors of interest using national health screening raw cohort data. Despite these limitations, our findings provide an empirical basis for measuring disease burden due to risk factors using PAFs and DALYs by analyzing data representative of an entire population. Comparing the magnitude of disease burden according to cancer type may provide meaningful data for health policy development and estimations of disease burden that can be reduced by alterations in health behaviors.

## 5. Conclusions

Our results suggest that in Koreans aged ≥40 years, simultaneous exposure to smoking and alcohol consumption is associated with higher cancer incidence and death rates than those associated with either of these factors; these associations are further mediated by sex and specific cancer site. The attributable disease burden may be further associated with age and occupation; this evidence may help develop interventions applicable to each sub-group. The presented methodology may be used in future studies examining the disease burden and effectiveness of interventions in various groups and disease types.

## Figures and Tables

**Table 1:** DALYs associated with 12 cancers in Koreans aged ≥40 years (2018) (unit: person-years).

Cancer Type	Men			Women		
	YLD	YLL	DALY	YLD	YLL	DALY
Esophageal	5745	11,180	16,925	632	2324	2956
Stomach	51,530	40,427	91,957	23,688	21,151	44,839
Liver	30,362	73,531	103,894	9305	21,873	31,178
Laryngeal	2778	2161	4939	157	404	561
Tracheal, bronchial, lung	32,424	87,126	119,550	18,675	35,126	53,802
Colon and rectal	40,280	38,553	78,834	25,694	28,269	53,963
Mouth	2896	3546	6442	1881	2080	3960
Nasopharyngeal	1212	1347	2560	353	558	912
Other pharyngeal or oropharyngeal parts	2918	2991	5909	412	634	1047
Gallbladder and biliary tract	6630	16,937	23,568	5155	15,365	20,519
Pancreatic	6172	25,682	31,854	4945	20,587	25,532
Bladder	14,142	5880	20,022	2772	2090	4862
Total	197,090	309,363	506,453	93,669	150,461	244,130

DALYs, disability-adjusted life years; YLDs, years lived with disability; YLLs, years of life lost.

**Table 2:** Cancer type-specific incidence and mortality rates stratified by smoking and alcohol consumption in Korean men.

Cancer Type	Combined Smoking and Alcohol Consumption		Smoking		Alcohol Consumption	
	Incidence	Mortality	Incidence	Mortality	Incidence	Mortality
	HR (95% CI)	HR (95% CI)	HR (95% CI)	HR (95% CI)	HR (95% CI)	HR (95% CI)
Esophageal	4.4 (2.7–7.3) *	6.1 (2.8–13.6) *	1.7 (1.2–2.5) *	2.3 (1.2–4.2) *	2.6 (1.8–3.8) *	2.7 (1.5–4.9) *
Stomach	1.3 (1.1–1.5)	1.3 (0.8–1.9)	1.2 (1.1–1.4)	1.4 (1.1–1.9)	1.1 (0.9–1.0)	0.9 (0.6–1.2)
Liver	1.6 (1.3-2.0) *	2.2 (1.5–3.2) *	1.2 (1.0–1.4) *	1.4 (1.1–1.9) *	1.3 (1.1–1.6) *	1.5 (1.1–2.1) *
Laryngeal	3.4 (1.9–6.3)	23.4 (1.7–313.4)	2.2 (1.4–3.4)	9.5 (1.1–84.9)	1.6 (1.0–2.5)	2.5 (0.5–12.5)
Tracheal, bronchial, and lung	2.3 (1.9–2.7) *	3.3 (2.5–4.4)	1.9 (1.7–2.2) *	2.8 (2.3–3.4)	1.2 (1.0–1.4) *	1.2 (1.0–1.5)
Colon and rectal	1.3 (1.1–1.6)	1.7 (1.0–2.8)	1.1 (0.9–1.2)	1.3 (0.9–1.9)	1.2 (1.1–1.4)	1.3 (0.9–1.9)
Mouth	1.4 (0.9–2.2)	0.7 (0.1–3.9)	1.1 (0.8–1.5)	0.8 (0.3–2.8)	1.3 (1.0–1.9)	0.9 (0.2–3.2)
Nasopharyngeal	1.6 (0.5–4.7)	2.2 (0.2–25.0)	1.0 (0.4–2.3)	1.3 (0.2–8.0)	1.6 (0.7–3.6)	1.7 (0.3–10.5)
Other parts of the pharynx and oropharynx	1.3 (0.6–2.8)	4.8 (0.6–36.8)	0.9 (0.5–1.5)	1.2 (0.3–5.7)	1.5 (0.9–2.7)	3.9 (0.9–18.2)
Gallbladder and biliary tract	1.4 (0.9–2.3)	1.2 (0.6–2.5)	1.1 (0.8–1.5)	1.3 (0.8–2.3)	1.4 (1.0–1.9)	0.9 (0.5–1.6)
Pancreatic	1.6 (1.1–2.5)	1.9 (1.1–3.3)	1.3 (1.0–1.8)	1.4 (0.9–2.1)	1.2 (0.9–1.7)	1.4 (0.9–2.1)
Bladder	1.4 (0.9–2.0)	1.8 (0.5–6.3)	1.3 (1.0–1.7)	1.6 (0.6–3.9)	1.0 (0.8–1.4)	1.2 (0.4–3.1)

Cause-specific Cox proportional hazard modeling was used. HR, hazard ratios; CI, confidence interval. * denotes statistically significant comparisons of ‘combined smoking and alcohol consumption’, ‘smoking’, and ‘alcohol consumption’.

**Table 3:** Cancer type-specific incidence and mortality rates stratified by smoking and alcohol consumption in Korean women.

Cancer Type	Combined Smoking and Alcohol Consumption		Smoking		Alcohol Consumption	
	Incidence	Mortality	Incidence	Mortality	Incidence	Mortality
	HR (95% CI)	HR (95% CI)	HR (95% CI)	HR (95% CI)	HR (95% CI)	HR (95% CI)
Esophageal	4.4 (2.6–7.3)	6.1 (2.8–13.6)	1.7 (1.2–2.5)	2.3 (1.2–4.2)	2.6 (1.8–3.8)	2.7 (1.5–4.9)
Stomach	1.2 (0.3–4.6)	1.2 (0.8–1.9)	1.5 (0.5–4.0)	1.4 (1.0–1.9)	0.8 (0.3–2.2)	0.9 (0.6–1.2)
Liver	**3.3 (1.0–10.7)**	2.1 (1.4–3.0)	2.3 (1.0–5.3)	1.4 (1.0–1.9)	1.5 (0.6–3.6)	1.5 (1.1–2.0)
Laryngeal	3.4 (1.8–6.2)	14.0 (1.4–134.9)	2.1 (1.4–3.3)	6.7 (1.1–41.4)	1.6 (1.0–2.5)	2.1 (0.4–10.1)
Tracheal, bronchial, and lung	**5.1 (2.0–13.0)**	**13.5 (2.6–69.1)**	**2.6 (1.3–5.2)**	**4.1 (1.1–14.7)**	2.0 (0.9–4.1)	3.3 (0.9–11.4)
Colon and rectal	1.6 (0.5–5.2)	1.9 (0.1–30.1)	0.9 (0.3–2.4)	1.3 (0.2–10.2)	1.8 (0.9–3.7)	1.5 (0.2–11.2)
Mouth	1.5 (0.3–9.2)	0.7 (0.1–3.9)	0.9 (0.2–3.7)	0.8 (0.3–2.8)	1.7 (0.5–5.5)	0.9 (0.2–3.2)
Nasopharyngeal	1.8 (0.6–5.3)	2.2 (0.2–25.0)	1.0 (0.4–2.2)	1.3 (0.2–8.0)	1.9 (0.9–4.2)	1.7 (0.3–10.5)
Other pharyngeal and oropharyngeal parts	1.2 (0.6–2.6)	4.9 (0.6–37.5)	0.9 (0.5–1.5)	1.2 (0.3–5.6)	1.5 (0.8–2.5)	4.1 (0.9–19.0)
Gallbladder and biliary tract	1.5 (0.9–2.3)	1.2 (0.6–2.5)	**3.3 (1.1–9.7)**	1.6 (0.2–12.9)	1.3 (0.9–1.8)	0.9 (0.5–1.6)
Pancreatic	**6.4 (1.3–31.4)**	4.4 (0.5–42.0)	2.7 (0.8–9.1)	**3.7 (1.0–13.5)**	2.4 (0.7–8.0)	1.2 (0.1–9.2)
Bladder	1.3 (0.9–1.9)	1.8 (0.5–6.0)	1.3 (1.0–1.7)	1.5 (0.6–3.7)	1.0 (0.8–1.4)	1.2 (0.4–3.0)

Cause-specific Cox proportional hazard modeling was used. HR, hazard ratios; CI, confidence interval. Bold values denote statistically significant findings (*p* < 0.05).

**Table 4:** PAFs for cancer in Korea according to sex.

Cancer Type	Incidence						Mortality					
	Combined Smoking and Alcohol Consumption		Smoking		Alcohol Consumption		Combined Smoking and Alcohol Consumption		Smoking		Alcohol Consumption	
	PAF (%)	95% CI	PAF (%)	95% CI	PAF (%)	95% CI	PAF (%)	95% CI	PAF (%)	95% CI	PAF (%)	95% CI
**Men, aged ≥40 years**												
Esophageal	25.5	(14.3–38.9)	18.0	(4.6–32.1)	26.7	(14.9–39.0)	34.0	(15.1–55.8)	28.7	(7.0–50.0)	27.8	(10.0–47.0)
Stomach	2.7	(0.8–4.9)	6.3	(2.2–10.4)	1.2	(−1.8 to 0.5)	2.6	(−1.8 to 8.7)	12.2	(1.9–23.0)	0.0	(0.0–0.0)
Liver	6.0	(3.0–9.4)	6.2	(0.8, 11.8)	7.3	(3.0–12.0)	10.4	(4.5–17.8)	11.4	(1.8–21.7)	10.7	(3.0–19.3)
Laryngeal	19.6	(8.1–34.7)	27.2	(10.9–43.4)	11.5	(−0.3 to 25.5)	69.2	(7.0–96.9)	72.9	(2.0–96.4)	24.9	(−13.2 to 72.2)
Tracheal, bronchial, and lung	11.3	(8.2–14.8)	22.7	(17.9–27.6)	3.8	(0.4–7.5)	18.8	(13.2–25.3)	35.9	(28.5–43.1)	4.2	(-0.8 to 9.8)
Colon and rectal	3.2	(1.1–5.6)	2.4	(−1.9 to 6.8)	5.0	(1.6–8.7)	6.3	(0.0–15.1)	8.3	(−3.9 to 21.6)	6.3	(−2.9 to 17.3)
Mouth	4.0	(−0.9 to 10.8)	1.6	(−8.5 to 12.8)	7.3	(−0.9 to 16.7)	0.0	(0.0–0.0)	0.0	(0.0–0.0)	0.0	(0.0–0.0)
Nasopharyngeal	5.6	(−4.8 to 26.9)	0.0	(0.0–0.0)	11.9	(−7.2 to 37.3)	10.4	(−8.9 to 70.7)	8.2	(−33 to 68.8)	13.4	(−19.8 to 68.3)
Other pharyngeal and oropharyngeal	3.3	(−3.8 to 15.4)	0.0	(0.0–0.0)	10.7	(−3.0 to 27.6)	27.7	(−3.9 to 78.3)	6.6	(−30.4 to 59.9)	40.0	(−3.5 to 79.6)
Gallbladder and biliary tract	4.2	(−0.9, 11.2)	1.8	(−8.5 to 13.4)	7.4	(−1.0 to 17.2)	1.9	(−4.6 to 13.3)	9.1	(−7.7 to 28.3)	0.0	(0.0–0.0)
Pancreatic	6.0	(0.7–13.1)	9.8	(−0.5 to 20.8)	4.7	(−3.0 to 13.6)	8.4	(1.0–18.9)	10.2	(−3.2 to 25.0)	8.4	(−1.9, 20.6)
Bladder	3.5	(−0.7, 8.9)	8.6	(−0.3, 18.1)	1.0	(−5.2 to 8.4)	7.6	(−5.0 to 34.6)	15.1	(−13.3 to 47.7)	3.6	(−14.4 to 31.8)
**Women, aged ≥40 years**												
Esophageal	1.2	(0.6–2.3)	1.6	(0.3–3.3)	6.2	(3.1–10.3)	1.9	(0.7–4.5)	2.8	(0.5–6.7)	6.6	(2.0–13.9)
Stomach	0.1	(−0.3 to 1.3)	1.0	(−1.1 to 6.5)	0.0	(0.0–0.0)	0.1	(−0.1 to 0.3)	0.9	(0.1–2.0)	0.0	(0.0–0.0)
Liver	0.9	(0.0–3.5)	2.8	(−0.1 to 9.0)	1.9	(−1.8 to 9.8)	0.4	(0.2–0.7)	0.9	(0.1–1.9)	2.0	(0.4–3.9)
Laryngeal	0.9	(0.3–1.9)	2.5	(0.8–5.0)	2.4	(0.0–6.0)	4.6	(0.2–33.3)	11.4	(0.2–47.9)	4.3	(−2.4 to 27.3)
Tracheal, bronchial, and lung	1.5	(0.4–4.3)	3.4	(0.6–8.8)	3.9	(-0.2 to 11.4)	4.4	(0.6–20.3)	6.6	(0.3–23.7)	8.6	(-0.2 to 30.2)
Colon and rectal	0.2	(−0.2 to 1.5)	0.0	(0.0–0.0)	3.3	(−0.5 to 10.2)	0.3	(−0.3 to 9.8)	0.7	(−1.9 to 17.3)	1.9	(−3.5 to 29.6)
Mouth	0.2	(−0.3 to 3.0)	0.0	(0.0–0.0)	2.8	(−2.0 to 15.7)	0.0	(0.0–0.0)	0.0	(0.0–0.0)	0.0	(0.0–0.0)
Nasopharyngeal	0.3	(−0.1 to 1.6)	0.0	(0.0–0.0)	3.5	(−0.6 to 11.6)	0.4	(−0.3 to 8.2)	0.6	(−1.8 to 13.7)	2.8	(−3.1 to 28.2)
Other pharyngeal and oropharyngeal parts	0.1	(−0.2 to 0.6)	0.0	(0.0–0.0)	1.9	(−0.7 to 6.0)	1.4	(−0.1 to 12.0)	0.4	(−1.7 to 9.4)	11.4	(−0.5 to 42.8)
Gallbladder and biliary tract	0.2	(0.0–0.5)	4.9	(0.2–16.5)	1.2	(−0.4 to 3.2)	0.1	(−0.2 to 0.5)	1.4	(−1.8 to 21.4)	0.0	(0.0–0.0)
Pancreatic	2.0	(0.1–10.2)	3.7	(−0.5 to 15.5)	5.5	(−1.2 to 22.6)	1.2	(−0.2 to 13.3)	5.8	(0.1–22.2)	0.7	(−3.7 to 25.5)
Bladder	0.1	(0.0 to 0.3)	0.6	(−0.1, 1.5)	0.2	(−0.9 to 1.6)	0.3	(−0.2 to 1.8)	1.2	(−0.9 to 5.9)	0.6	(−2.4 to 7.7)

PAF, population attributable fraction; CI, confidence interval.

**Table 5:** Burden of cancers attributable to smoking and alcohol consumption according to sex (unit: person-years).

Cancer Type	DALYs Attributable to Combined Smoking and Alcohol Consumption (A)		DALYs Attributable to Smoking (B)		DALYs Attributable to Alcohol Consumption (C)	
**Men, aged ≥40 years**						
**All sites**	**48,306**	(9.5)	75,117	(14.8)	30,934	(6.1)
Esophageal	5273	(31.2)	4239	(25.0)	4645	(27.4)
Stomach	2434	(2.6)	8138	(8.8)	613	(0.7)
Liver	9464	(9.1)	10,302	(9.9)	10,115	(9.7)
Laryngeal	2042	(41.3)	2329	(47.2)	858	(17.4)
Tracheal, bronchial, lung	20,081	(16.8)	38,617	(32.3)	4908	(4.1)
Colon and rectal	3708	(4.7)	4143	(5.3)	4459	(5.7)
Mouth	116	(1.8)	45	(0.7)	211	(3.3)
Nasopharyngeal	209	(8.2)	110	(4.3)	326	(12.7)
Other pharyngeal or oropharyngeal parts	925	(15.6)	197	(3.3)	1509	(25.5)
Gallbladder and biliary tract	596	(2.5)	1664	(7.1)	492	(2.1)
Pancreatic	2519	(7.9)	3232	(10.1)	2438	(7.7)
Bladder	939	(4.7)	2100	(10.5)	361	(1.8)
**Women, aged ≥40 years**						
All sites	2666	(1.1)	6080	(2.5)	6599	(2.7)
Esophageal	52	(1.7)	75	(2.5)	192	(6.5)
Stomach	34	(0.1)	443	(1.0)	0	(0.0)
Liver	166	(0.5)	455	(1.5)	603	(1.9)
Laryngeal	20	(3.6)	50	(8.9)	21	(3.8)
Tracheal, bronchial, lung	1841	(3.4)	2959	(5.5)	3760	(7.0)
Colon and rectal	154	(0.3)	197	(0.4)	1365	(2.5)
Mouth	4	(0.1)	0	(0.0)	53	(1.3)
Nasopharyngeal	4	(0.4)	4	(0.4)	28	(3.1)
Other pharyngeal or oropharyngeal parts	9	(0.9)	3	(0.3)	80	(7.6)
Gallbladder and biliary tract	19	(0.1)	468	(2.3)	59	(0.3)
Pancreatic	354	(1.4)	1385	(5.4)	418	(1.6)
Bladder	9	(0.2)	41	(0.8)	18	(0.4)

DALYs, disability-adjusted life years.

## Data Availability

Data are available on reasonable request. All data are open for access and analysis, contingent upon the approval of the National Health Insurance Data Sharing Service. Applications to use NHIS claim data are reviewed by the inquiry committee of research support and, once approved, raw data—deidentified to preserve the privacy of research participants—are provided to the applicant at a fee. Researchers can access claims data by using a statistical analysis tool at the ‘Big Data Analysis Center’, where a personal computer that can read and analyze claim data is installed. Researchers from outside the country can only gain access to the data by conducting a joint study with Korean researchers. To reuse claim data, researchers must obtain the approval of the NHIS and pay an additional fee before the study period expires.

## Supplementary Materials

The following supporting information can be downloaded at: https://www.mdpi.com/article/10.3390/ijerph19063493/s1, Table S1: Effects of smoking and alcohol consumption on cancer incidence and mortality risk in Korean men; Table S2: Effects of smoking and alcohol consumption on cancer incidence and mortality risk in Korean women.
