# Supplementary material for: Burden of Cancer Due to Cigarette Smoking and Alcohol Consumption in Korea
Source: Int J Environ Res Public Health. 2022 Mar 15;19(6):3493. doi: 10.3390/ijerph19063493 (PMC8950006; doi:10.3390/ijerph19063493)
Supplement: Supplementary file 1 [file ijerph-19-03493-s001.zip › ijerph-1609547-supplementary.pdf]

Table S1. Effects of smoking and alcohol consumption on cancer incidence and mortality risk in Korean men

Cancer type	Combined smoking and alcohol consumption				Smoking				Alcohol consumption			
	Incidence		Mortality		Incidence		Mortality		Incidence		Mortality	
	Control	Case	Control	Case	Control	Case	Control	Case	Control	Case	Control	Case
Esophageal												
Number of cases	35	32	13	17	57	52	20	25	55	54	21	24
Follow-up (person-years)	278,398	49,103	278,506	49,183	340,718	155,088	340,893	155,229	384,383	111,423	384,552	111,570
Multivariate HR (95% CI)	Ref	4.4 (2.7–7.3)	Ref	6.1 (2.8–13.6)	Ref	1.7 (1.2–2.5)	Ref	2.3 (1.2–4.2)	Ref	2.6 (1.8–3.8)	Ref	2.7 (1.5–4.9)
Stomach												
Number of cases	587	147	89	23	740	418	110	73	858	300	139	44
Follow-up (person-years)	275,047	48,523	277,715	49,101	336,595	153,168	339,950	154,947	379,693	110,071	383,561	111,336
Multivariate HR (95% CI)	Ref	1.3 (1.1–1.5)	Ref	1.3 (0.8–1.9)	Ref	1.2 (1.1–1.4)	Ref	1.4 (1.1–1.9)	Ref	1.1 (0.9–1.0)	Ref	0.9 (0.6–1.2)
Liver												
Number of cases	311	99	87	41	408	238	117	85	450	196	131	71
Follow-up (person-years)	277,039	48,807	278,049	49,144	338,975	154,285	340,337	155,032	382,516	110,743	383,937	111,431
Multivariate HR (95% CI)	Ref	1.6 (1.3-2.0)	Ref	2.2 (1.5–3.2)	Ref	1.2 (1.0–1.4)	Ref	1.4 (1.1–1.9)	Ref	1.3 (1.1–1.6)	Ref	1.5 (1.1–2.1)
Laryngeal												
Number of cases	29	19	1	3	39	40	1	5	50	29	3	3

Follow-up (person-years)	278,224	49,111	278,392	49,182	340,585	155,020	340,780	155,194	384,134	111,472	384,404	111,571
Multivariate HR (95% CI)	Ref	3.4 (1.9–6.3)	Ref	23.4 (1.7–313.4)	Ref	2.2 (1.4–3.4)	Ref	9.5 (1.1–84.9)	Ref	1.6 (1.0–2.5)	Ref	2.5 (0.5–12.5)
Tracheal, bronchial, and lung												
Number of cases	356	156	121	79	471	441	171	230	641	271	272	129
Follow-up (person-years)	276,923	48,700	278,103	49,071	338,880	153,905	340,401	154,932	382,129	110,656	383,963	111,369
Multivariate HR (95% CI)	Ref	2.3 (1.9–2.7)	Ref	3.3 (2.5–4.4)	Ref	1.9 (1.7–2.2)	Ref	2.8 (2.3–3.4)	Ref	1.2 (1.0–1.4)	Ref	1.2 (1.0–1.5)
Colon and rectal												
Number of cases	508	120	54	16	670	333	75	46	721	282	84	37
Follow-up (person-years)	275,358	48,600	277,600	49,102	336,851	153,516	339,768	154,889	380,273	110,094	383,387	111,270
Multivariate HR (95% CI)	Ref	1.3 (1.1–1.6)	Ref	1.7 (1.0–2.8)	Ref	1.1 (0.9–1.2)	Ref	1.3 (0.9–1.9)	Ref	1.2 (1.1–1.4)	Ref	1.3 (0.9–1.9)
Mouth												
Number of cases	78	26	6	-	104	56	9	4	108	52	10	3
Follow-up (person-years)	277,798	48,962	278,310	49,153	339,924	154,750	340,608	155,101	383,585	111,089	384,258	111,451
Multivariate HR (95% CI)	Ref	1.4 (0.9–2.2)	Ref	0.7 (0.1–3.9)	Ref	1.1 (0.8–1.5)	Ref	0.8 (0.3–2.8)	Ref	1.3 (1.0–1.9)	Ref	0.9 (0.2–3.2)
Nasopharyngeal												
Number of cases	13	4	3	2	18	9	3	2	18	9	3	2

Follow-up (person-years)	278,501	49,173	278,550	49,183	340,877	155,205	340,961	155,242	384,532	111,549	384,610	111,594
Multivariate HR (95% CI)	Ref	1.6 (0.5–4.7)	Ref	2.2 (0.2–25.0)	Ref	1.0 (0.4–2.3)	Ref	1.3 (0.2–8.0)	Ref	1.6 (0.7–3.6)	Ref	1.7 (0.3–10.5)
Other parts of the pharynx and oropharynx												
Number of cases	29	8	2	2	40	18	4	3	39	19	3	4
Follow-up (person-years)	278,422	49,149	278,561	49,183	340,782	155,139	340,961	155,221	384,412	111,509	384,595	111,557
Multivariate HR (95% CI)	Ref	1.3 (0.6–2.8)	Ref	4.8 (0.6–36.8)	Ref	0.9 (0.5–1.5)	Ref	1.2 (0.3–5.7)	Ref	1.5 (0.9–2.7)	Ref	3.9 (0.9–18.2)
Gallbladder and biliary tract												
Number of cases	72	18	32	7	102	52	40	22	106	48	47	15
Follow-up (person-years)	278,293	49,131	278,489	49,170	340,596	155,101	340,891	155,218	384,263	111,435	384,537	111,573
Multivariate HR (95% CI)	Ref	1.4 (0.9–2.3)	Ref	1.2 (0.6–2.5)	Ref	1.1 (0.8–1.5)	Ref	1.3 (0.8–2.3)	Ref	1.4 (1.0–1.9)	Ref	0.9 (0.5–1.6)
Pancreatic												
Number of cases	86	27	45	16	110	67	62	39	126	51	68	33
Follow-up (person-years)	278,293	49,133	278,500	49,182	340,681	155,144	340,917	155,242	384,304	111,521	384,560	111,600
Multivariate HR (95% CI)	Ref	1.6 (1.1–2.5)	Ref	1.9 (1.1–3.3)	Ref	1.3 (1.0–1.8)	Ref	1.4 (0.9–2.1)	Ref	1.2 (0.9–1.7)	Ref	1.4 (0.9–2.1)
Bladder												
Number of cases	121	29	12	5	155	86	13	8	178	63	15	6

Follow-up (person-years)	277,845	49,067	278,304	49,175	340,073	154,724	340,668	155,079	383,501	111,296	384,208	111,539
Multivariate HR (95% CI)	Ref	1.4 (0.9–2.0)	Ref	1.8 (0.5–6.3)	Ref	1.3 (1.0–1.7)	Ref	1.6 (0.6–3.9)	Ref	1.0 (0.8–1.4)	Ref	1.2 (0.4–3.1)

Cause-specific Cox proportional hazard modeling was used.

CI, confidence interval; HR, hazard ratio; Ref, reference.

Table S2. Effects of smoking and alcohol consumption on cancer incidence and mortality risk in Korean women

Cancer type	Combined smoking and alcohol consumption				Smoking				Alcohol consumption			
	Incidence		Mortality		Incidence		Mortality		Incidence		Mortality	
	Control	Case	Control	Case	Control	Case	Control	Case	Control	Case	Control	Case
Esophageal												
Number of cases	37	32	13	17	59	52	20	25	57	54	21	24
Follow-up (person-years)	397,105	49,570	397,221	49,649	464,222	157,886	464,405	158,028	505,421	116,688	505,599	116,834
Multivariate HR (95% CI)	Ref	4.4 (2.6–7.3)	Ref	6.1 (2.8–13.6)	Ref	1.7 (1.2–2.5)	Ref	2.3 (1.2–4.2)	Ref	2.6 (1.8–3.8)	Ref	2.7 (1.5–4.9)
Stomach												
Number of cases	103	-	104	23	107	4	125	73	107	4	154	44
Follow-up (person-years)	117,993	467	396,206	49,568	122,768	2,785	463,238	157,745	120,311	5,242	504,383	116,600
Multivariate HR (95% CI)	Ref	1.2 (0.3–4.6)	Ref	1.2 (0.8–1.9)	Ref	1.5 (0.5–4.0)	Ref	1.4 (1.0–1.9)	Ref	0.8 (0.3–2.2)	Ref	0.9 (0.6–1.2)
Liver												
Number of cases	76	1	104	41	80	6	134	85	81	5	148	71
Follow-up (person-years)	118,297	458	396,613	49,611	123,079	2,775	463,698	157,830	120,612	5,242	504,832	116,695
Multivariate HR (95% CI)	Ref	3.3 (1.0–10.7)	Ref	2.1 (1.4–3.0)	Ref	2.3 (1.0–5.3)	Ref	1.4 (1.0–1.9)	Ref	1.5 (0.6–3.6)	Ref	1.5 (1.1–2.0)
Laryngeal												
Number of cases	31	19	2	3	41	40	2	5	52	29	4	3

Follow up (person-years)	396,927	49,577	397,106	49,650	464,086	157,819	464,292	157,992	505,169	116,736	505,448	116,836
Multivariate HR (95% CI)	Ref	3.4 (1.8–6.2)	Ref	14.0 (1.4–134.9)	Ref	2.1 (1.4–3.3)	Ref	6.7 (1.1–41.4)	Ref	1.6 (1.0–2.5)	Ref	2.1 (0.4–10.1)
Tracheal, bronchial, and lung												
Number of cases	89	2	17	1	95	9	19	3	96	8	19	3
Follow-up (person-years)	118,219	453	118,591	456	122,982	2,760	123,378	2,788	120,526	5,216	120,923	5,244
Multivariate HR (95% CI)	Ref	5.1 (2.0–13.0)	Ref	13.5 (2.6–69.1)	Ref	2.6 (1.3–5.2)	Ref	4.1 (1.1–14.7)	Ref	2.0 (0.9–4.1)	Ref	3.3 (0.9–11.4)
Colon and rectal												
Number of cases	108	-	16	-	116	4	17	1	112	8	17	1
Follow-up (person-years)	118,052	467	118,551	467	122,804	2,781	123,339	2,798	120,367	5,218	120,883	5,255
Multivariate HR (95% CI)	Ref	1.6 (0.5–5.2)	Ref	1.9 (0.1–30.1)	Ref	0.9 (0.3–2.4)	Ref	1.3 (0.2–10.2)	Ref	1.8 (0.9–3.7)	Ref	1.5 (0.2–11.2)
Mouth												
Number of cases	49	2	6	-	50	2	9	4	49	3	10	3
Follow-up (person-years)	118,083	447	396,777	49,620	122,860	2,767	463,861	157,888	120,403	5,223	504,887	116,613
Multivariate HR (95% CI)	Ref	1.5 (0.3–9.2)	Ref	0.7 (0.1–3.9)	Ref	0.9 (0.2–3.7)	Ref	0.8 (0.3–2.8)	Ref	1.7 (0.5–5.5)	Ref	0.9 (0.2–3.2)
Nasopharyngeal												
Number of cases	14	4	3	2	20	9	3	2	19	10	3	2

Follow-up (person-years)	397,205	49,640	397,255	49,649	464,371	158,003	464,464	158,041	505,568	116,805	505,646	116,858
Multivariate HR (95% CI)	Ref	1.8 (0.6–5.3)	Ref	2.2 (0.2–25.0)	Ref	1.0 (0.4–2.2)	Ref	1.3 (0.2–8.0)	Ref	1.9 (0.9–4.2)	Ref	1.7 (0.3–10.5)
Other pharyngeal and oropharyngeal parts												
Number of cases	36	8	2	2	47	18	4	3	46	19	3	4
Follow up (person-years)	397,097	49,616	397,276	49,650	464,254	157,937	464,474	158,019	505,419	116,774	505,646	116,847
Multivariate HR (95% CI)	Ref	1.2 (0.6–2.6)	Ref	4.9 (0.6–37.5)	Ref	0.9 (0.5–1.5)	Ref	1.2 (0.3–5.6)	Ref	1.5 (0.8–2.5)	Ref	4.1 (0.9–19.0)
Gallbladder and biliary tract												
Number of cases	101	18	45	7	29	4	13	1	139	48	61	15
Follow up (person-years)	396,920	49,598	397,161	49,637	123,425	2,796	123,470	2,799	505,220	116,700	505,541	116,838
Multivariate HR (95% CI)	Ref	1.5 (0.9–2.3)	Ref	1.2 (0.6–2.5)	Ref	3.3 (1.1–9.7)	Ref	1.6 (0.2–12.9)	Ref	1.3 (0.9–1.8)	Ref	0.9 (0.5–1.6)
Pancreatic												
Number of cases	26	-	16	-	29	3	17	3	29	3	19	1
Follow-up (person-years)	118,659	467	118,716	467	123,453	2,797	123,514	2,798	120,990	5,261	121,047	5,264
Multivariate HR (95% CI)	Ref	6.4 (1.3–31.4)	Ref	4.4 (0.5–42.0)	Ref	2.7 (0.8–9.1)	Ref	3.7 (1.0–13.5)	Ref	2.4 (0.7–8.0)	Ref	1.2 (0.1–9.2)
Bladder												
Number of cases	133	29	13	5	167	86	14	8	190	63	16	6

Follow-up (person-years)	396,488	49,534	396,999	49,641	463,514	157,522	464,161	157,878	504,476	116,561	505,234	116,803
Multivariate HR (95% CI)	Ref	1.3 (0.9–1.9)	Ref	1.8 (0.5–6.0)	Ref	1.3 (1.0–1.7)	Ref	1.5 (0.6–3.7)	Ref	1.0 (0.8–1.4)	Ref	1.2 (0.4–3.0)

Cause-specific Cox proportional hazard modeling was used.

CI, confidence interval; HR, hazard ratio; Ref, reference.
